# Supplementary material for: Spin density wave and van Hove singularity in the kagome metal CeTi3Bi4
Source: Nat Commun. 2025 May 12;16:4384. doi: 10.1038/s41467-025-59460-4 (PMC12069633; doi:10.1038/s41467-025-59460-4)
Supplement: Supplementary file 1 — Supplementary Information [file 41467_2025_59460_MOESM1_ESM.pdf]

Supplementary Information: Spin density wave and van Hove singularity in the kagome metal $CeTi_3Bi_4$

Pyeongjae Park[1†], Brenden R. Ortiz[1], Milo Sprague[2], Anup Pradhan Sakhya[2], Si Athena Chen[3], Matthias. D. Frontzek[3], Wei Tian[3], Romain Sibille[4], Daniel G. Mazzone[4], Chihiro Tabata[5,6], Koji Kaneko[5,6], Lisa M. DeBeer-Schmitt[3], Matthew B. Stone[3], David S. Parker[1], German D. Samolyuk[1], Hu Miao[1], Madhab Neupane[2], & Andrew D. Christianson[1†]

[1]*Materials Science and Technology Division, Oak Ridge National Laboratory, Oak Ridge, TN 37831, USA*

[2]*Department of Physics, University of Central Florida, Orlando, Florida 32816, USA*

[3]*Neutron Scattering Division, Oak Ridge National Laboratory, Oak Ridge, Tennessee 37831, USA*

[4]*PSI Center for Neutron and Muon Sciences, 5232 Villigen PSI, Switzerland*

[5]*Materials Sciences Research Center, Japan Atomic Energy Agency, Tokai, Ibaraki 319-1195, Japan*

[6]*Advanced Science Research Center, Japan Atomic Energy Agency, Tokai, Ibaraki 319-1195, Japan*

[†]Corresponding author: parkp@ornl.gov, christiansad@ornl.gov

Supplementary Note 1. Conventional and primitive orthorhombic cell convention for the FCC lattice.

All diffraction data in this study are referenced to the reciprocal lattice of the conventional orthorhombic unit cell (Fig. 1a), which consist of the following lattice vectors:

$$\mathbf{a} = a\hat{x}, \qquad \mathbf{b} = b\hat{y}, \qquad \mathbf{c} = c\hat{z}, \tag{1}$$

where a, b, and c are lattice parameters. This convention yields the following orthogonal reciprocal lattice vectors:

$$\mathbf{a}^* = \frac{2\pi}{a}\hat{x}, \qquad \mathbf{b}^* = \frac{2\pi}{b}\hat{y}, \qquad \mathbf{c}^* = \frac{2\pi}{c}\hat{z}. \tag{2}$$

We express the cartesian coordinate as (x, y, z) from hereafter, e.g., **a** = (a, 0, 0). All neutron diffraction data in this work are described based on this convention, e.g., $\mathbf{Q}_{\mathrm{IC}}$ = (0, 0.94, 0) (r.l.u.) = 0.94$\mathbf{b}^*$. Meanwhile, a standard choice of the lattice vectors for the primitive orthorhombic face-centered cubic (FCC) lattice is:

$$\mathbf{a}_1 = (0, \frac{b}{2}, \frac{c}{2}), \qquad \mathbf{a}_2 = (\frac{a}{2}, 0, \frac{c}{2}), \qquad \mathbf{a}_3 = (\frac{a}{2}, \frac{b}{2}, 0), \tag{3}$$

which yield corresponding non-orthogonal reciprocal lattice vectors:

$$\mathbf{b}_1 = 2\pi\left(-\frac{1}{a}, \frac{1}{b}, \frac{1}{c}\right), \qquad \mathbf{b}_2 = 2\pi\left(\frac{1}{a}, -\frac{1}{b}, \frac{1}{c}\right), \qquad \mathbf{b}_3 = 2\pi\left(\frac{1}{a}, \frac{1}{b}, -\frac{1}{c}\right). \tag{4}$$

The two different sets in Supplementary Eqs. (2) and (4) are related as follows:

$$\mathbf{a}^* = \frac{\mathbf{b}_2 + \mathbf{b}_3}{2}, \qquad \mathbf{b}^* = \frac{\mathbf{b}_1 + \mathbf{b}_3}{2} \qquad \mathbf{c}^* = \frac{\mathbf{b}_1 + \mathbf{b}_2}{2}. \tag{5}$$

The X and Y high-symmetry points in the first Brillouin zone of the primitive FCC convention [Supplementary Eq. (4)] is simply $\mathbf{a}^*$ and $\mathbf{b}^*$, respectively. Thus, $\mathbf{Q}_{\mathrm{c}}$ = (0, 1, 0) (r.l.u.) observed in our neutron diffraction data lies on the Y point, which corresponds to the M points of the pseudo-hexagonal framework (see Fig. 4a in the main text). In other words, $\mathbf{Q}_{\mathrm{c}}$ is identical to the **G**/2, where **G** represents a reciprocal lattice vector of a hexagonal lattice, and $\mathbf{Q}_{\mathrm{IC}} = 0.94\mathbf{Q}_{\mathrm{c}}$ is nearly equivalent to **G**/2.

Supplementary Note 2. Assignment of the incommensurate ordering wave vector $\mathbf{Q}_{\mathrm{IC}}$.

The incommensurate Bragg peaks only appear near the commensurate magnetic reflections (i.e., near $\mathbf{Q} = \mathbf{Q}_{\mathrm{nuc}} + \mathbf{Q}_{\mathrm{C}}$), suggesting that the correct ordering wave vector for this component is $\mathbf{Q}_{\mathrm{IC}}$ = (0, ±0.94, 0) (r.l.u.), rather than (0, ±0.06, 0). If it were the latter, primary satellite reflections would appear around the nuclear reflections at $\mathbf{Q} = \mathbf{Q}_{\mathrm{nuc}}$ + (0, ±0.06, 0), which were not observed. Fig. 2f illustrates the diffraction pattern with the two correctly assigned ordering wave vectors. For instance, the incommensurate peaks at (0, 1.06, 2) and at (0, 0.94, 2) correspond to satellite reflections from $\mathbf{Q}_{\mathrm{nuc}}$ = (0, 2, 2) and (0, 0, 2), respectively.

Supplementary Note 3. Size of ordered magnetic moments from neutron diffraction data refinement.

Our least-square refinement of the neutron diffraction data also provides the size of ordered moments for each modulation, $\mathbf{Q}_{\mathrm{C}}$ and $\mathbf{Q}_{\mathrm{IC}}$. However, this requires a two-fold analysis, as we obtain different results based on two scenarios: (i) the double-**Q** scenario and (ii) phase separation of $\mathbf{Q}_{\mathrm{c}}$ and $\mathbf{Q}_{\mathrm{IC}}$. Determining which scenario is correct cannot be resolved by our results presented in this work, as mentioned in the main text.

The double-**Q** scenario assumes that the entire crystal contributes to both $\mathbf{Q}_{\mathrm{C}}$ and $\mathbf{Q}_{\mathrm{IC}}$, with the resultant magnetic structure being a linear combination of the two spin configurations (Fig. 2j and 2k). The refined moments for this case are shown in Supplementary Table 4. The moment values of $\mathbf{Q}_{\mathrm{IC}}$ in this table represent the maximum amplitude of the SDW modulation, and the averaged moment amplitude per Ce^{3+} site is $2/\pi \simeq 0.637$ times the values presented in Supplementary Table 4. In the phase separation scenario, the refined ordered moments depend on the domain fraction of $\mathbf{Q}_{\mathrm{C}}$ and $\mathbf{Q}_{\mathrm{IC}}$. This can be denoted by f_{C} and f_{IC}, which range between 0 and 1 and satisfies $f_{\mathrm{C}} + f_{\mathrm{IC}} = 1$. Since this scenario assumes only a fraction of the sample contributes to each modulation's Bragg peak, the fitted ordered magnetic moment increases accordingly to match the observed intensity. The increase factor is $1/\sqrt{f_{\mathrm{c}}}$ and $1/\sqrt{f_{\mathrm{IC}}}$, respectively, as structure factor is proportional to the moment squared. For instance, if $\mathbf{Q}_{\mathrm{c}}$ and $\mathbf{Q}_{\mathrm{IC}}$ domains are equally populated ($f_{\mathrm{C}} = f_{\mathrm{IC}} = 0.5$), the refined ordered moment is $\sqrt{2}$ times the values shown in Supplementary Table 4.

Supplementary Note 4. Assessing the degree of Kondo effects in Ce^{3+} magnetism.

Ce^{3+} based magnets are well known for exhibiting heavy-fermion behavior, which could be another potential source of strongly correlated physics in $CeTi_3Bi_4$, in addition to the effects arising from Ti kagome electronic structure. Thus, we carefully assessed whether $CeTi_3Bi_4$ can be categorized as a heavy-fermion system, which would affect how we interpret our observations. We concluded that Kondo effects in $CeTi_3Bi_4$ are marginal compared to typical heavy-fermion Ce^{3+} systems, based on the following observations:

First, the integrated magnetic entropy reaches Rln2–the expected saturation value for a low-lying Kramers doublet–around T_{N} (Fig. 1d). In heavy-fermion systems with significant Kondo temperatures, the restoration of the full magnetic entropy (Rln2) is usually achieved at temperatures much higher than T_{N}, which contrasts with our observation. Second, the analysis of the Sommerfeld coefficient, γ, reveals a value markedly smaller than that found in Ce-based heavy-fermion systems. To estimate γ, we fitted the low-temperature part of the heat capacity using the following equation[1,2]:

$$C_{\mathrm{p}} = \gamma T + c\Delta_{\mathrm{SW}}^{7/2}\sqrt{T}\,\mathrm{e}^{\frac{\Delta_{\mathrm{SW}}}{T}} \times \left[1 + \frac{39}{20}\frac{T}{\Delta_{\mathrm{SW}}} + \frac{51}{32}\left(\frac{T}{\Delta_{\mathrm{SW}}}\right)^2\right], \quad (6)$$

where C_{p} is the heat capacity, T is temperature, Δ_{SW} is the spin-wave energy gap, and c is a coefficient

related to D in the long-wavelength magnon dispersion $\omega(\mathbf{k}) = \sqrt{\Delta_{\mathrm{SW}}^2 + D|\mathbf{k}|^2}$ with $c \propto D^{-3}$ [1,2]. The complex second term compared to the usual T^3 behavior for bosonic excitations (magnons) was used because the magnon dispersion in $CeTi_3Bi_4$ should exhibit a sizable energy gap due to easy-axis anisotropy along the **b**-axis (Fig. 1f), requiring consideration of thermal population activation. The best-fitted curve, shown as orange lines in Supplementary Fig. 1, gives γ = 35(2) mJ mol^{-1} K^{-2}, Δ_{SW} = 7.43(3) K, and c = 33.9(1) mJ/mol K^4. The fitted γ value is much less significant than values typically found in heavy-fermion systems Ce^{3+} systems ($\gamma \simeq$ 100 mJ mol^{-1} K^{-2} or higher)[3,4].

To better assess the significance of Kondo effects in $CeTi_3Bi_4$, we compared its low-temperature heat capacity to that of $LaTi_3Bi_4$, a non-magnetic analogue lacking potential correlation effects associated with Ce^{3+} ions. As shown in Supplementary Fig. 1, fitting the data with the model $C(T) = \gamma T + AT^3$ yields γ = 10.2(1) mJ mol^{-1} K^{-2} for $LaTi_3Bi_4$, which is approximately 3.4 times smaller than that of $CeTi_3Bi_4$. While this result suggests that some degree of electron correlation is present in $CeTi_3Bi_4$ –which is not surprising for Ce^{3+} systems–the γ value of $CeTi_3Bi_4$ does not exhibit an order-of-magnitude enhancement compared to the uncorrelated case of $LaTi_3Bi_4$. This indicates correlation effects in $CeTi_3Bi_4$ are not large enough to be considered the dominant factor in determining its physical properties.

Finally, the magnitude of the ordered moment derived from least-square refinement (see Supplementary Note 3) indicates strong magnetic order in $CeTi_3Bi_4$, in contrast to the weak or absent magnetic order typically observed in heavy-fermion systems. For both the double-**Q** and phase separation scenarios, the ordered moment is close to the fully saturated moment observed in the *M*–*H* curve (Fig. 1f), which stands in stark contrast with the weak long-range order seen in Kondo systems.

Supplementary Note 5. Predominance of RKKY mechanism in driving the $\mathbf{Q}_{\mathrm{IC}}$ and $\mathbf{Q}_{\mathrm{C}}$ modulations.

As mentioned in the main text, an RKKY mechanism generally governs the magnetic interactions in Ce-based metallic systems through conduction electrons. Here, we provide a more specific supporting argument for the case of $\mathbf{Q}_{\mathrm{c}}$ and $\mathbf{Q}_{\mathrm{IC}}$ modulations in $CeTi_3Bi_4$, based on a qualitative analysis from the perspective of magnetic exchange interactions.

First, the ferromagnetic alignment along the Ce^{3+} chains and out-of-plane directions indicates net ferromagnetic interactions along those axes, such as $J_{||}$ and J_c in Fig. 2j. Notably, the Curie-Weiss temperature of $CeTi_3Bi_4$ (θ_{CW} = 3 K) being nearly identical to T_{N} = 3.4 K [5] means these ferromagnetic interactions dominate the system, likely through intra-chain interactions (e.g., $J_{||}$) given the layered nature of this compound implying much weaker interactions along the c-axis[6].

While the ferromagnetic alignment within each Ce^{3+} chain is firmly stabilized by the strongest intra-chain interactions, the observed inter-chain spin modulations–the key feature of the observed magnetic ground states–arise from subdominant antiferromagnetic inter-chain couplings. The antiferromagnetic alignment between nearest-neighbor (NN) Ce^{3+} spin chains (Figs. 2j and 2k) indicates net antiferromagnetic interactions between them (e.g., antiferromagnetic $J_{\perp 1}$ in Fig. 1b). However, stabilizing the incommensurate inter-chain modulation $\mathbf{Q}_{\mathrm{IC}}$ necessitates additional magnetic interactions between further-NN Ce^{3+} chains that give rise to exchange frustration, such as antiferromagnetic $J_{\perp 2}$ in Fig. 1b. Importantly, these inter-chain interactions occur over significant bond lengths (≥ 5.9 Å), making direct or super/double-exchange mechanisms unlikely (Fig. 1b). Thus, while

such mechanisms may contribute to short-range intra-chain interactions to some extent [7], the inter-chain interaction profile responsible for the SDW modulation is likely determined predominantly by the RKKY interaction mediated by conduction electrons.

Supplementary Note 6. Tempearature-dependent correlation lengths of the $\mathbf{Q}_{IC}$ and $\mathbf{Q}_{C}$ modulations.

Supplementary Fig. 8 presents the temperature dependence of the inverse full width at half maximum (FWHM) for the (0, δ, -10) and (0, 1, -10) magnetic Bragg peaks multiplied by 2π (see Figs. 3a–d). In the absence of instrumental resolution effects, this quantity would serve as a direct measure of the correlation length for each modulation. However, the instrumental resolution significantly contributes to the measured FWHM. The instrumental resolution estimated by a FWHM of nearby nuclear peaks, such as (0, 2, -10), is nearly the same as that of the magnetic peaks at the base temperatures ($\simeq$ 0.011 $Å^{-1}$). This is further confirmed by the rocking curves shown in Supplementary Fig. 2, which exhibit nearly the same angular width for both nuclear and magnetic peaks. These observations indicate that both the commensurate and incommensurate magnetic peaks are nearly resolution-limited.

As a result, the actual correlation lengths of the magnetic peaks are expected to be significantly longer than the values inferred from Supplementary Fig. 8. In such a case, attempting to correct for resolution effects through simple deconvolution would introduce substantial systematic errors. Therefore, instead of estimating absolute correlation lengths, we present the original $2\pi \times (\mathrm{FWHM})^{-1}$ values in Supplementary Fig. 8 and focus on the qualitative temperature dependence only.

The temperature dependence provides further insight into the nature of the co-existing magnetic modulations in $CeTi_3Bi_4$ below T_2. First, while no noticeable change in FWHM is observed above T_2 = 3.0 K, the correlation length of the SDW modulation gradually increases below this temperature, coinciding with the onset of the commensurate spin modulation. Second, as the temperature decreases further below T_2, the FWHM values (and thus the correlation lengths) of the commensurate and incommensurate phases become markedly similar. Together with the temperature dependence of $\delta(T)$ shown in Fig. 3d, these observations imply that the $\mathbf{Q}_C$ and $\mathbf{Q}_{IC}$ modulations do not behave independently.

Supplementary Note 7. Nature of the α band observed in ARPES.

In this note, we provide more explanation for the α band observed in ARPES but not in our DFT calculations. To better understand its nature, we further analyzed data taken at several incident photon energies: 50, 55, 60, 65, and 70 eV (the datasets presented in the main text were collected at 65 eV). Supplementary Fig. 13a–b shows the photon energy dependence of the α band, which exhibits almost no variation with photon energy, indicating that it is nearly dispersionless along the out-of-plane momentum (k_z) direction.

Meanwhile, it is important to note that a small electron pocket around the $\bar{\Gamma}$ point, resembling the α band, has been consistently observed in other materials from the $Ln$$Ti_3Bi_4$ family. For instance, see Refs. [5] and [8] of the Supplementary Information. Indeed, our DFT calculations predict an electron-like band centered at the $\bar{\Gamma}$ point, which is slightly above E_F ($E = E_F + 0.33$ eV) at $\bar{\Gamma}$ but forms an electron pocket at $k_z = \pi$, as indicated by the red dashed circles in Supplementary Fig. 13c. This is a strong candidate for the observed α band.

Nevertheless, despite its possible bulk origin described above, the α band observed in ARPES is very likely to possess a strong surface character. Unlike the experimentally observed α band, our DFT calculations suggest substantial dispersion of this band along the k_z direction (the right panel of Supplementary Fig. 13c). Such dispersive behavior of the $\bar{\Gamma}$-centered pocket is found in most band structures of the $Ln\mathrm{Ti_3Bi_4}$ family, which is attributed to its *p*-orbital character.

Thus, the α band is either a pure surface state or, a bulk-derived state that becomes localized at the surface. In both scenarios, the α band is strongly influenced by surface effects and cannot be considered an ideal bulk state.

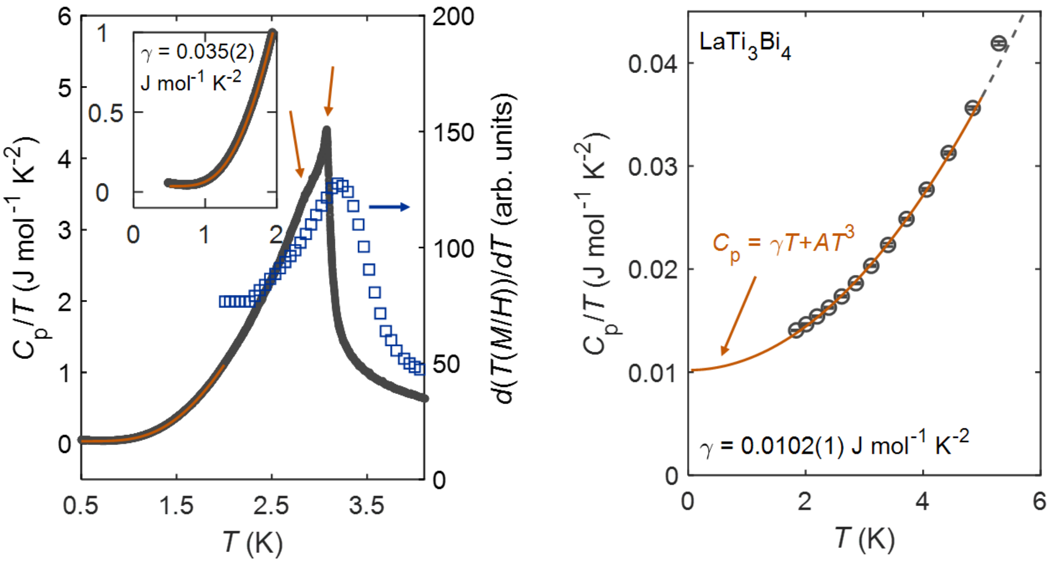

Supplementary Fig. 1 | Detailed heat capacity analysis of $CeTi_3Bi_4$ and $LaTi_3Bi_4$ around T_N and a base temperature. (Left) Heat capacity data collected using the ^{3}He insert option (see Methods). The orange solid lines represent the calculated values based on Supplementary Eq. (6) in the Supplementary Notes. We also overlay the first derivative of the M/H multiplied by temperature ($T \times (M/H)$, see Fig. 1e for measured M), a quantity used to identify the transition temperature in analogy to heat capacity[9]. The estimated transition temperature T_N derived from these two quantities 3.15 K and 3.24 K, well within a reasonable agreement range. **(Right)** Heat capacity of $LaTi_3Bi_4$, a non-magnetic analogue of $CeTi_3Bi_4$. The temperature range spanned by the dashed grey line was excluded from the phenomenological fitting of $C_p = \gamma T + AT^3$. Error bars represent the standard deviation of the measured heat capacity.

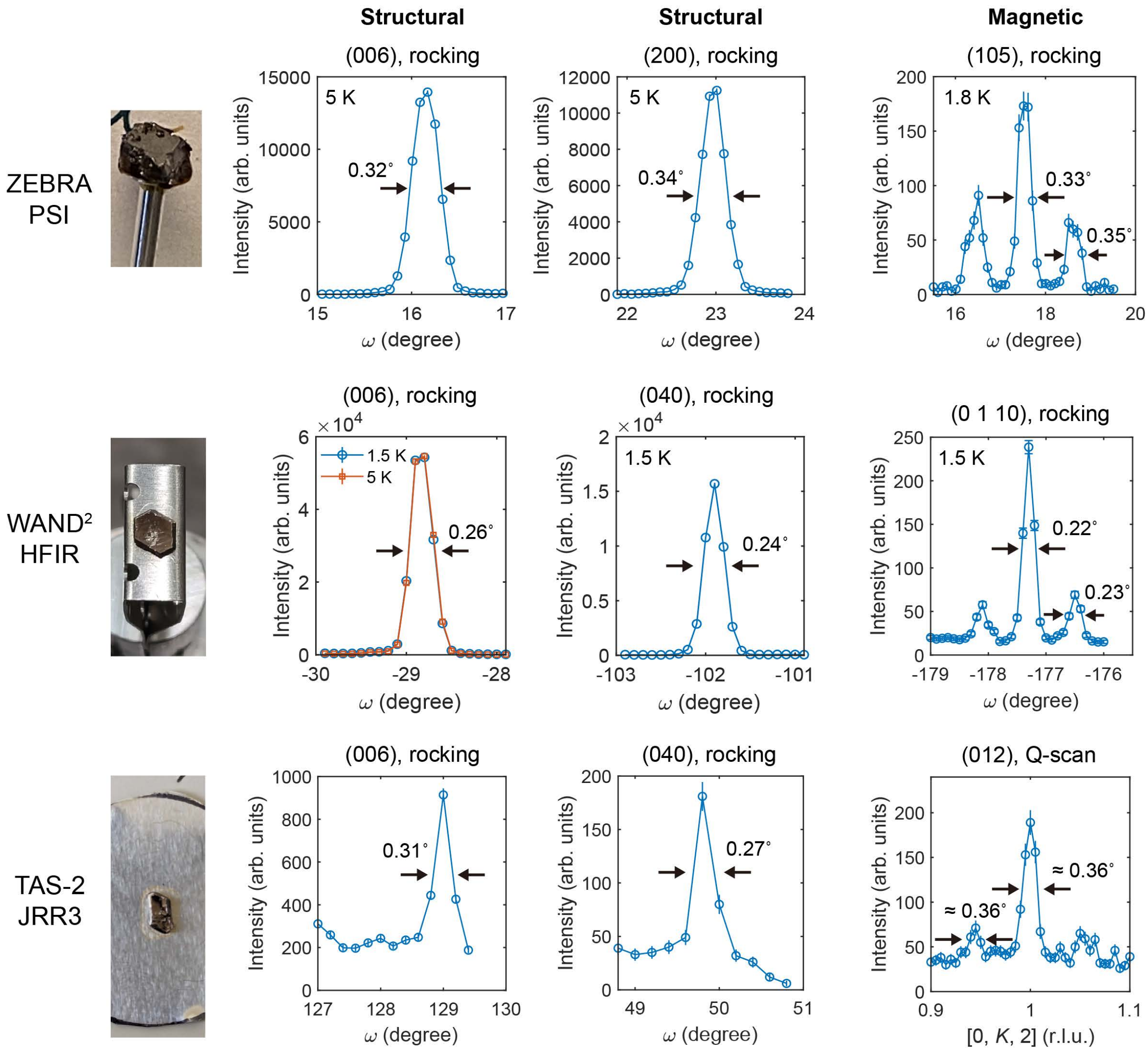

Supplementary Fig. 2 | Three crystals and corresponding rocking curves of nuclear and magnetic Bragg peaks measured at three different neutron beamlines. The angle values in each panel indicate the full width at half maximum (FWHM) of the respective peaks. For the nuclear peaks measured at ZEBRA and WAND^2, error bars (= standard deviations) are much smaller than the data symbols. All samples exhibit near-resolution-limited FWHM, confirming high sample quality and ruling out extrinsic factors as the source of the observed commensurate and incommensurate magnetic reflections. For the magnetic reflection measurements at TAS-2, a rocking scan was not performed; instead, a [0, *K*, 0] scan is shown in the bottom-right panel. The approximate FWHM in azimuthal rotation angle (ω) was estimated based on its relation to the **b*** vector and may not be directly comparable to the FWHM values obtained from rocking scans of the nuclear peaks.

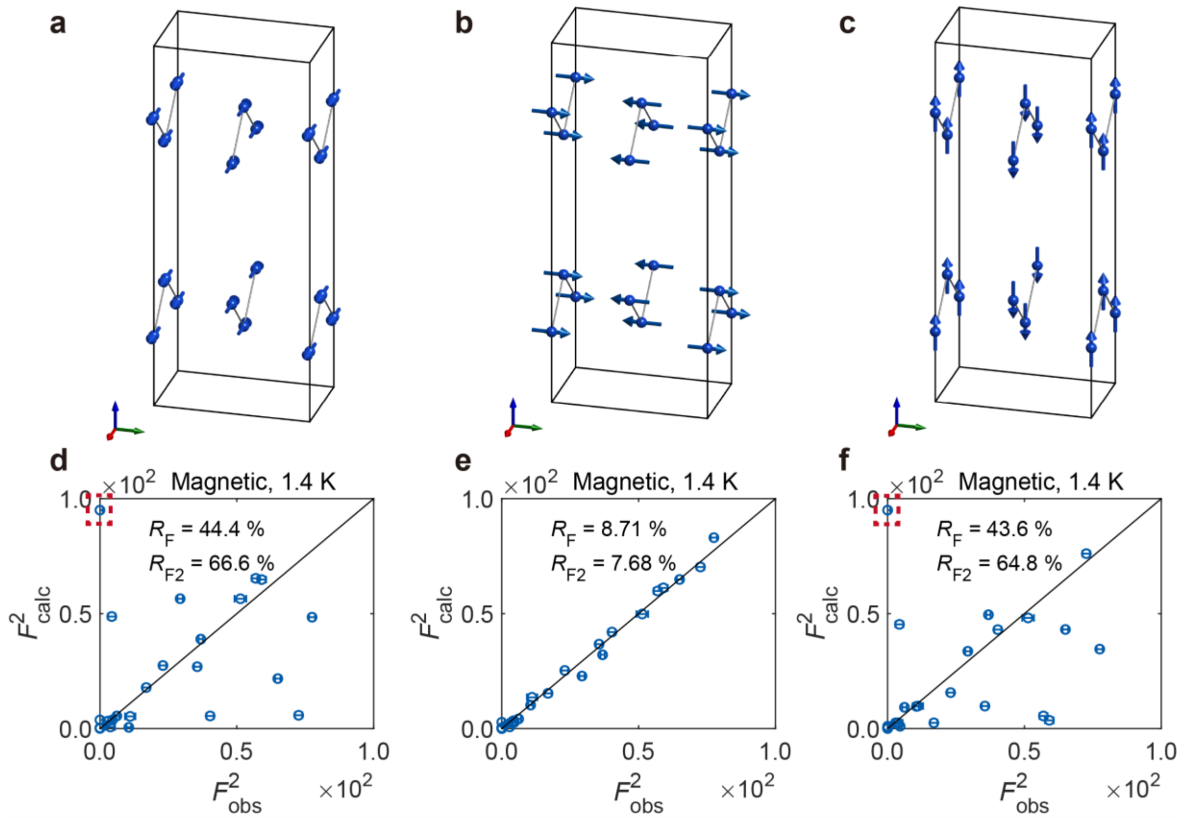

Supplementary Fig. 3 | Comparison of the best-fit magnetic structure associated with Q_C to other potential models. Panels **d**–**f** show observed versus simulated neutron diffraction intensities (F^2) for the magnetic structure models in **a**–**c**, where **b** corresponds to the best-fit model (Fig. 2j). The red dashed boxes in **d** and **f** highlight the sizable simulated intensity of the (0, 1, 0) peak, in contrast to its absence in the data. Error bars represent the standard deviation of the integrated intensity.

Supplementary Table 1 | Crystal structure obtained from the least-square refinement of the ZEBRA data. See Fig. 2g in the main text.

T = 1.5 K	Space group: *F m m m* (No. 69)<br>Cell dimensions: a = 5.935 Å, b = 10.374 Å c = 25.067 Å, $\alpha = \beta = \gamma = 90°$			
Atom	Wyckoff positions	x/*a*	y/*b*	z/*c*
Bi1	8*i*	1	0.5	0.5684(3)
Bi2	16*m*	0	0.1615(5)	0.3128(2)
Bi3	8*h*	0.5	0.3311(7)	0.5
Ce1	8*i*	0	0.5	0.6959(4)
Ti1	16*j*	0.25	0.25	0.4050(4)
Ti2	8*i*	0.5	0.5	0.5915(6)
	Overall B factor: 0.01 Å^2			
	Agreement factors: R_F (%) = 4.67, R_{F2} (%) = 7.98, χ^2 = 21.4			

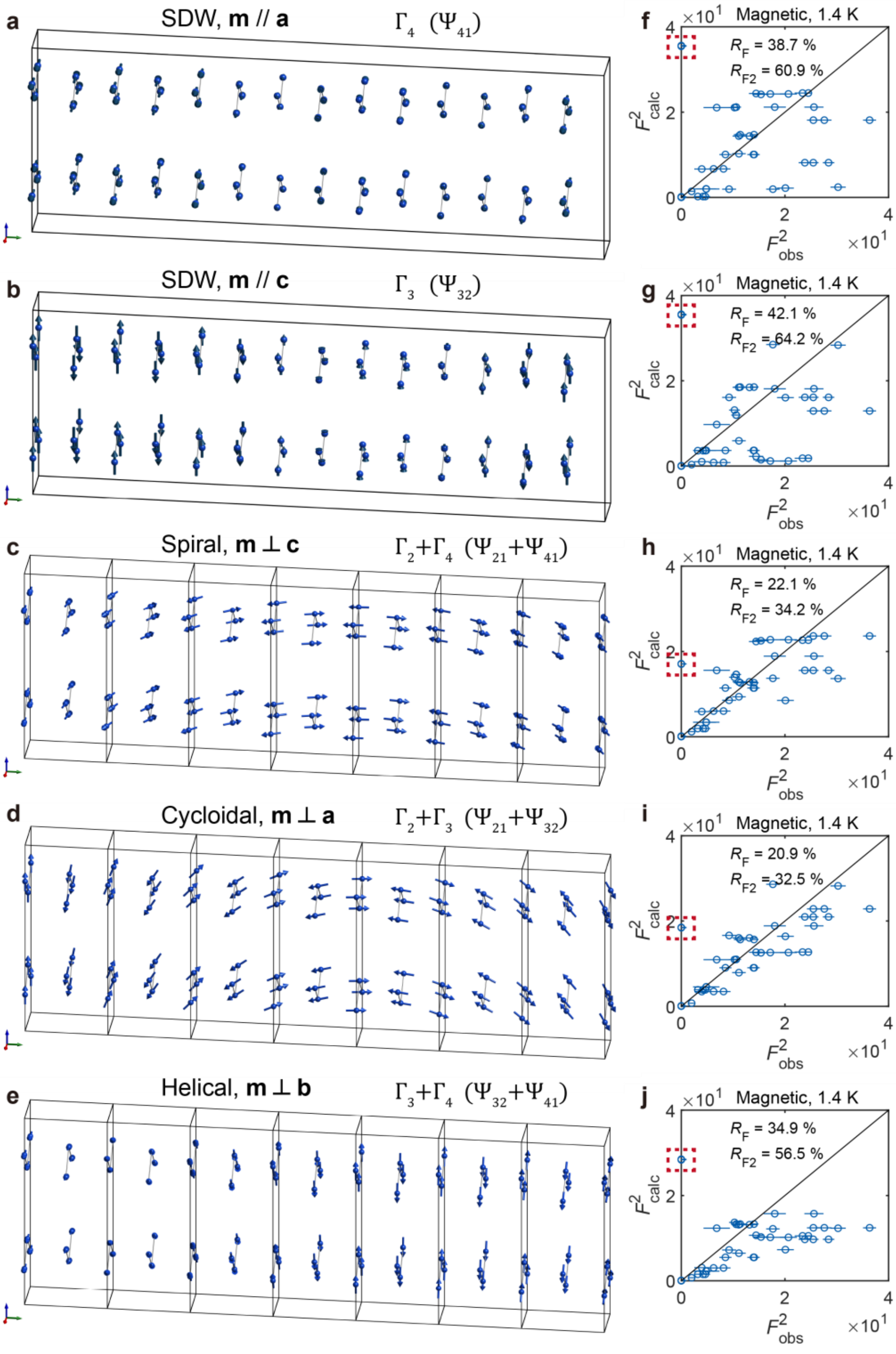

Supplementary Fig. 4 | Observed and simulated intensities (F^2) of incommensurate magnetic reflections (Q_{IC}) from various <u>incorrect</u> magnetic structure models. a–e, Incorrect magnetic structure models considered, which yield worse agreement factors (R_F and R_{F2} in **f–j**, respectively). The red dashed boxes in **f–j** highlight the sizable simulated intensity of the (0, 0.94, 0) and (0, 1.06, 0) peaks, in contrast to their absence in the data. The irreducible representations and basis vectors of each structure model are detailed in Supplementary Tables 2–3. of All alternative models show significantly worse agreement with the data compared to our solution presented in Fig. 2k. The best-fit magnetic structure model can be found in Fig. 2k. Error bars represent the standard deviation of the integrated intensity.

Supplementary Table 2. Character table of the *Fmmm* space group with the magnetic ordering wavevector $\mathbf{Q}_{IC}$ = (0, 0.94, 0). C_{2i} and m_i (i = a, b, c) represent the two-fold rotation about the i-axis and the mirror operation with a plane perpendicular to the i-axis, respectively.

	1 (x, y, z)	C_{2b} (-x, y, -z)	m_c (x, y, -z)	m_a (-x, y, z)
Γ_1	1	1	1	1
Γ_2	1	1	-1	-1
Γ_3	1	-1	1	-1
Γ_4	1	-1	-1	1

Supplementary Table 3. Basis vectors of the four irreducible representations (Irreps) in Supplementary Table 2 for $\mathbf{Q}_{IC}$ = (0, 0.94, 0). The parameter α can be found in the crystal structure refinement results (Supplementary Table 1).

Irreps	Basis vectors	Ce1 (0, 1/2, 1-α)	Ce2 (0, 1/2, α)
Γ_1	$\boldsymbol{\psi}_{11}$	(1, 0, 0)	(-1, 0, 0)
Γ_2	$\boldsymbol{\psi}_{21}$	(0, 1, 0)	(0, 1, 0)
	$\boldsymbol{\psi}_{22}$	(0, 0, 1)	(0, 0, -1)
Γ_3	$\boldsymbol{\psi}_{31}$	(0, 1, 0)	(0, -1, 0)
	$\boldsymbol{\psi}_{32}$	(0, 0, 1)	(0, 0, 1)
Γ_4	$\boldsymbol{\psi}_{41}$	(1, 0, 0)	(1, 0, 0)

Supplementary Table 4. Refined ordered moments from the ZEBRA data (Fig. 2h–i) and $WAND^2$ data (see Supplementary Fig. 6 and 10), assuming a double-**Q** magnetic structure (see Supplementary Notes).

Dataset	$\mathbf{Q}_C$	$\mathbf{Q}_{IC}$
ZEBRA, PSI<br>T = 1.5 K	$0.862(8)\mu_B/Ce^{3+}$<br>(24 reflections)	$1.05(1)\mu_B/Ce^{3+}$<br>(47 reflections)
$WAND^2$, HFIR<br>T = 1.5 K	$0.73(4)\mu_B/Ce^{3+}$<br>(11 reflections)	$1.02(6)\mu_B/Ce^{3+}$<br>(12 reflections)
$WAND^2$, HFIR<br>T = 2.6 K, H = 2.5 T	N/A	$0.79(5)\mu_B/Ce^{3+}$
$WAND^2$, HFIR<br>T = 3.1 K	N/A	$0.72(4)\mu_B/Ce^{3+}$

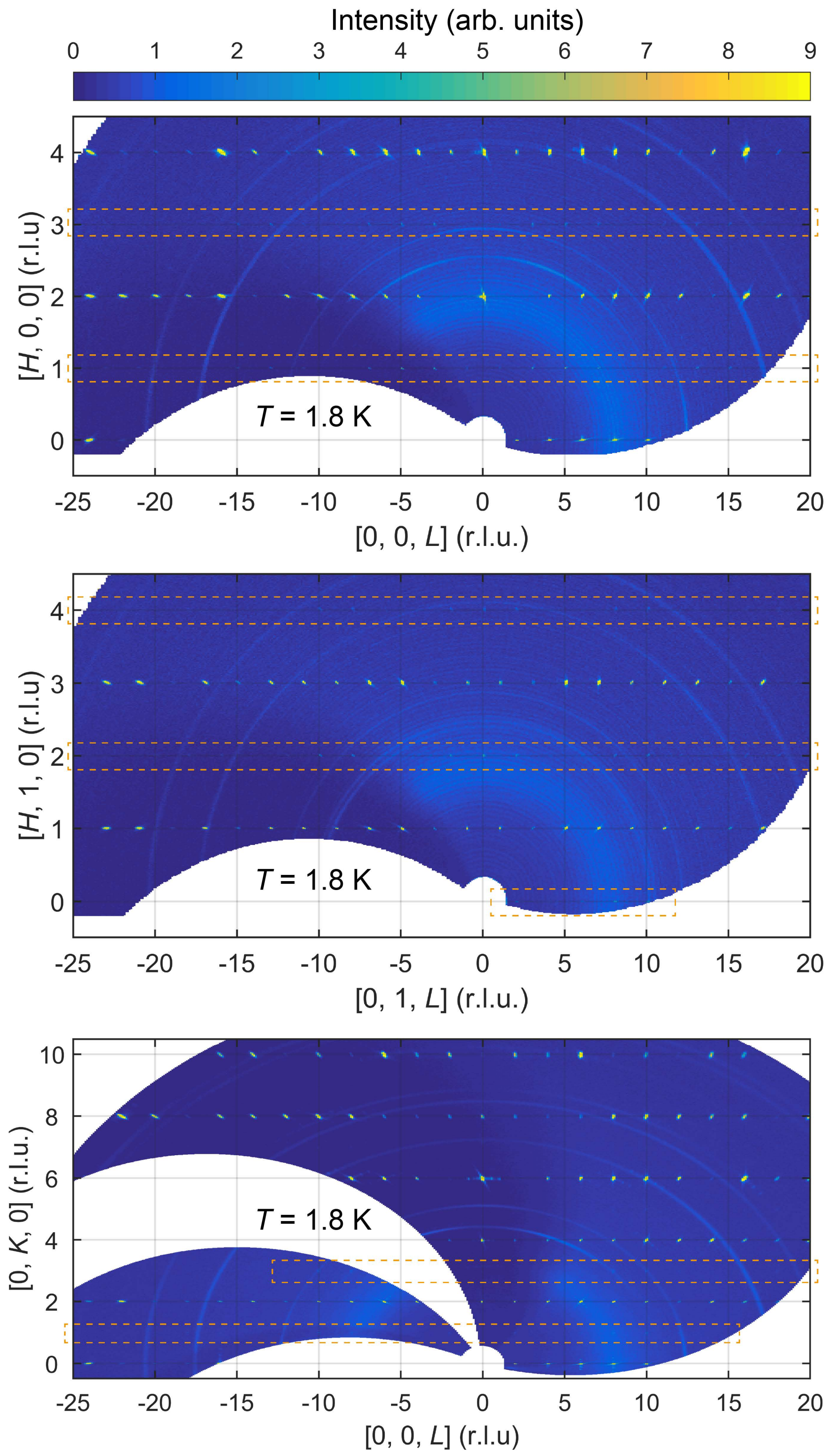

Supplementary Fig. 5 | Full neutron diffraction profiles of magnetically ordered $CeTi_3Bi_4$ at 1.8 K, measured at WAND². Orange dashed boxes highlight the magnetic reflections at nuclear-forbidden reciprocal coordinates. All spectra show that the magnetic reflections weaken at large-enough *H, K,* and *L*, consistent with the behavior expected from a magnetic form factor.

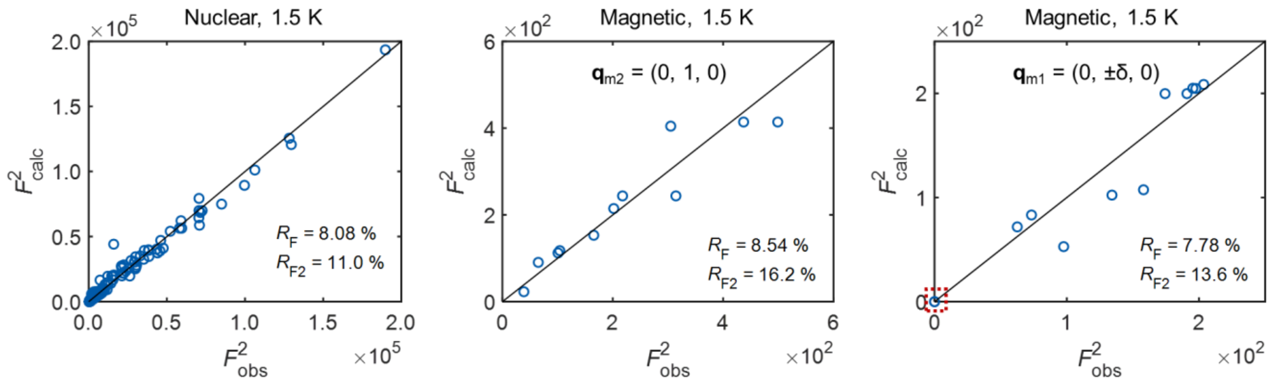

Supplementary Fig. 6 | Least-square refinement analysis results for the nuclear and magnetic reflections collected at WAND^2. The resulting crystal structure is provided in Supplementary Table 5. Magnetic structure models are shown in Figs. 2j–k of the main text.

Supplementary Table 5 | Crystal structure obtained from the least-square refinement of the WAND^2 data, as shown in the leftmost panel of Supplementary Fig. 6.

T = 1.5 K	Space group: *F m m m* (No. 69)<br>Cell dimensions: a = 5.919(1) Å, b = 10.308(1) Å c = 24.978(2) Å, $\alpha = \beta = \gamma = 90°$				
Atom	Wyckoff positions	x/a	y/b	z/c	B_{iso} (Å^2)
Bi1	8*i*	1	0.5	0.5673(2)	0.01
Bi2	16*m*	0	0.1616(6)	0.3126(1)	0.01
Bi3	8*h*	0.5	0.3310(9)	0.5	0.01
Ce1	8*i*	0	0.5	0.6951(4)	0.01
Ti1	16*j*	0.25	0.25	0.4063(4)	0.24(16)
Ti2	8*i*	0.5	0.5	0.5922(6)	0.20(22)
	Agreement factors: R_F (%) = 8.08, R_{F2} (%) = 11.0, χ^2 = 90.8				

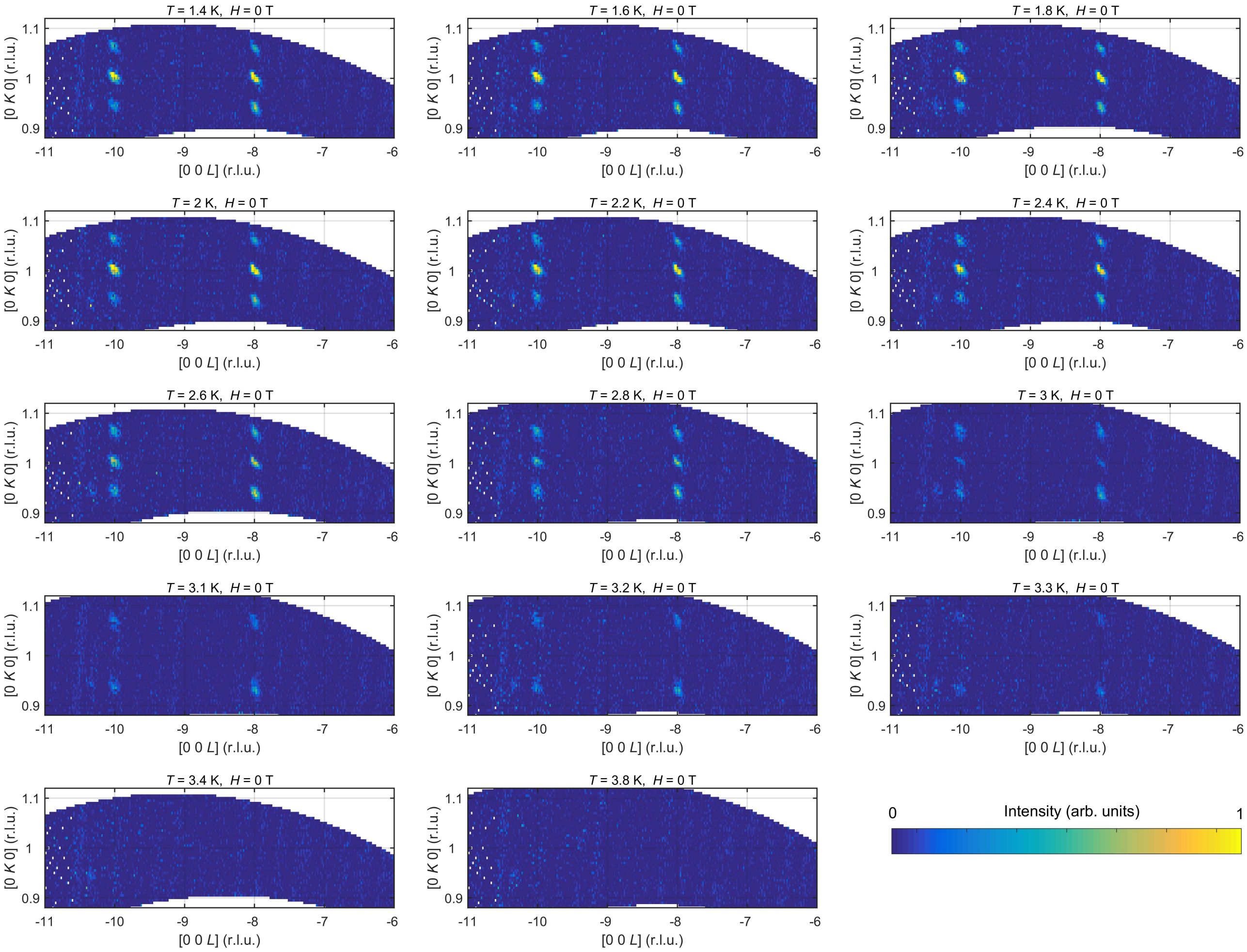

Supplementary Fig. 7 | Full temperature dependence dataset for the two magnetic reflections (Q_c and Q_{IC}) in $CeTi_3Bi_4$. No external magnetic fields were applied. The data shown in this figure were collected at WAND[2].

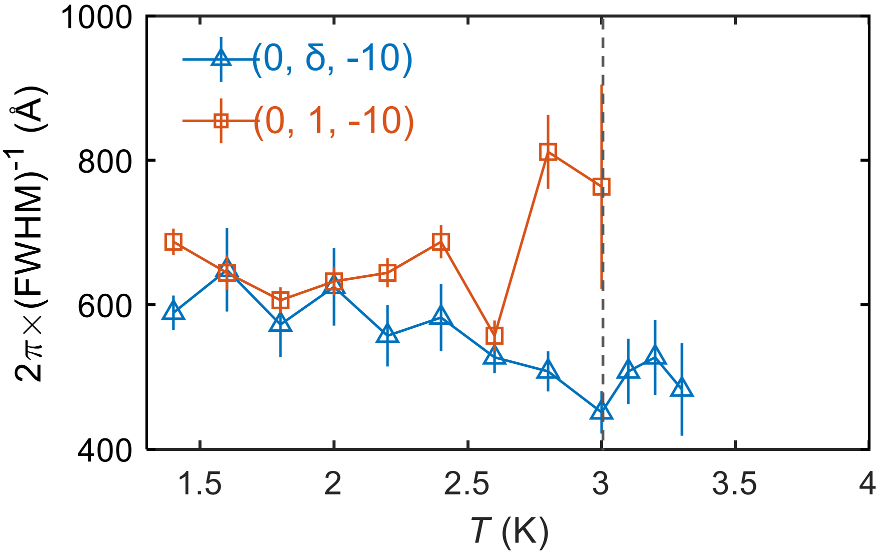

Supplementary Fig. 8 | Temperature dependence of the inverse full width at half maximum (FWHM) for the commensurate and incommensurate magnetic Bragg peaks along the [0, *K,* 0] direction. We fitted the data shown in Supplementary Fig. 7. Neglecting instrumental resolution, this quantity corresponds to the correlation length of the incommensurate spin-density wave (SDW) and the commensurate antiferromagnetic order. However, when considering the instrumental resolution's contribution to the fitted FWHM–which is challenging to quantity accurately–the actual correlation lengths of both modulations are expected to be much longer than the values shown here. Error bars represent the uncertainties in the fitted FWHM.

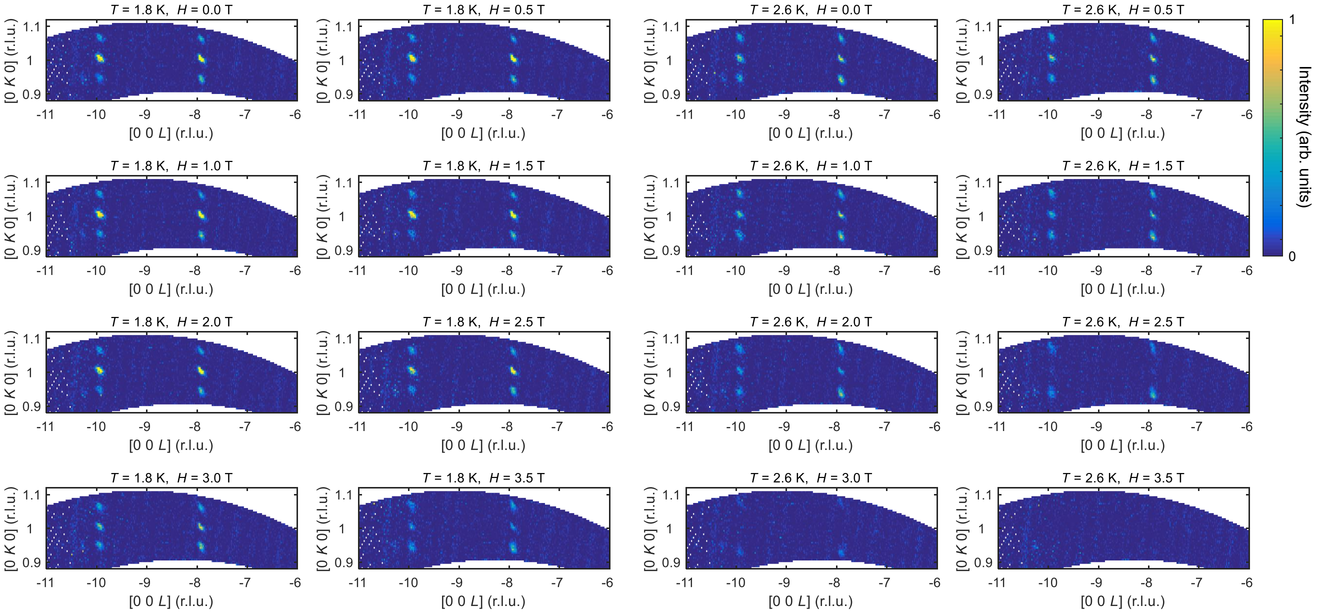

Supplementary Fig. 9 | Full field dependence dataset (H // a) for the two magnetic reflections (Q_c and Q_{IC}) in $CeTi_3Bi_4$ at T = 1.8 and 2.6 K. The data shown in this figure were collected at $WAND^2$.

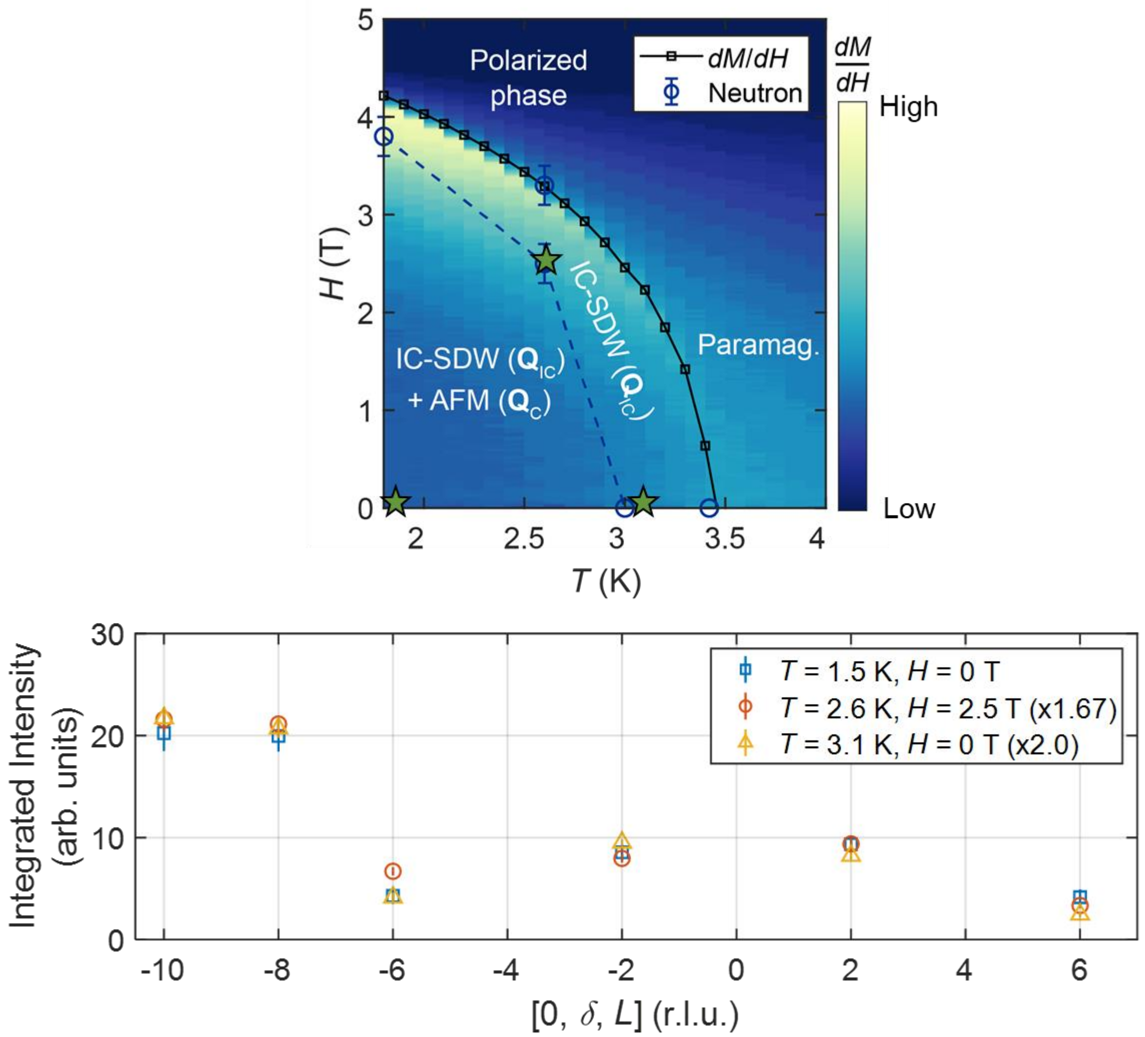

Supplementary Fig. 10 | Robustness of the spin density wave (SDW) ground state across the temperature-field phase diagram. Incommensurate magnetic reflections ($\mathbf{Q}_{IC}$) collected at three distinct temperature and field points (indicated by green star symbols) exhibit nearly identical intensity profiles (lower panel), suggesting a consistent SDW spin configuration throughout the entire phase diagram. For clarity, the overall intensity scale for the 2.6 K (orange) and 3.1 K (yellow) data has been adjusted. The neutron diffraction data shown in this figure were collected at WAND^2. Error bars represent the uncertainties in the integrated intensity.

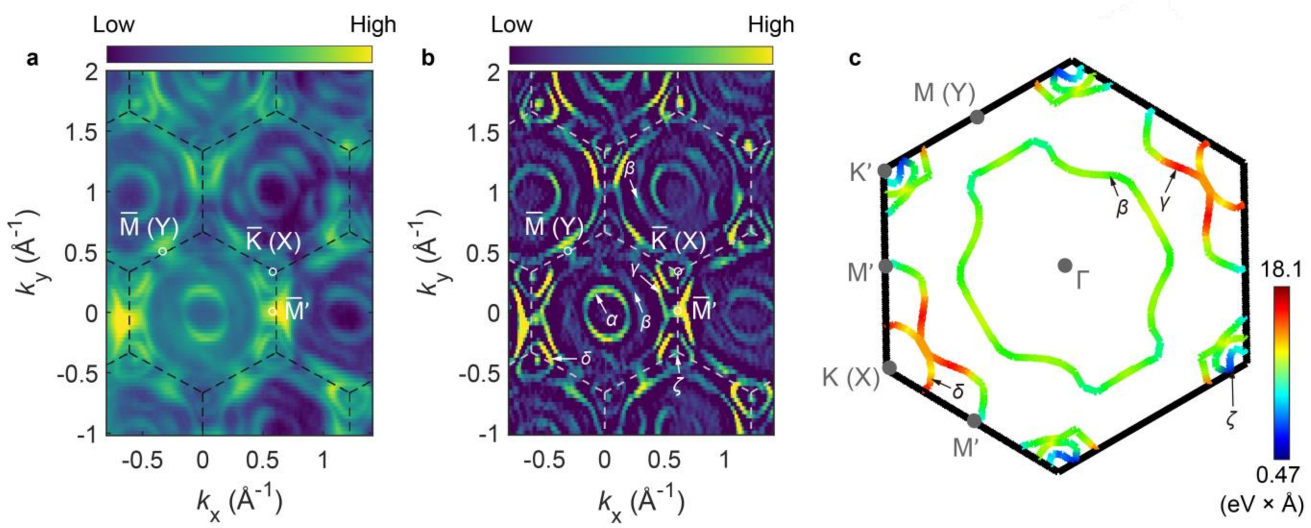

Supplementary Fig. 11 | Detailed comparison of the Fermi surface from ARPES and DFT. a–b, ARPES spectra at $E = E_F$, shown using the measured intensity and its second-derivative. **c**, Fermi-surface cross-section from the DFT calculations. The color scale indicates the Fermi velocity (eV × Å). Most of the electronic structure (β, γ, δ, and ζ bands) matches between ARPES and DFT, except for the electron pocket around the Γ point (α) not present in the DFT results. We conjecture that the α band observed in ARPES is a surface state.

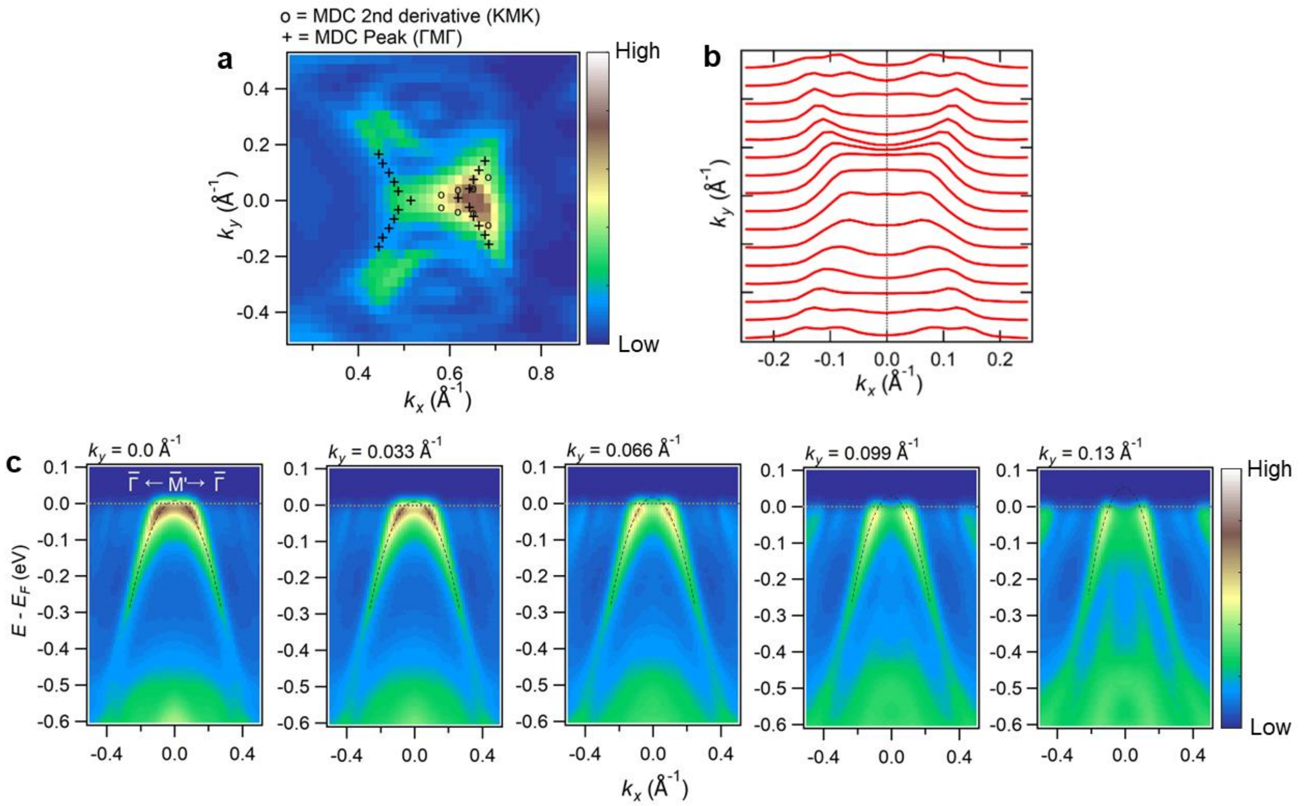

Supplementary Fig. 12 | Detailed analysis of the band structure around the $\bar{\mathrm{M}}'$ point. a–b Quantitative analysis of the Fermi surface shape near the van Hove singularity at the $\bar{\mathrm{M}}'$ point. The "+" and "o" symbols mark the momentum positions where the dispersion crosses the Fermi energy, extracted from fitting of the momentum distribution curves (MDCs) of the photoemission intensity and its second derivative along the two principal momentum directions. The color scale in **a** represents photoemission intensity, while **b** shows the same intensity distribution but symmetrized with respect to the $\overline{\mathrm{KM'K}}$ line. **c**, Measured ARPES band dispersion along the $\overline{\Gamma\mathrm{M}'}$ direction at different k_y values (see **a**). The dispersion along the k_x direction ($\mathbf{k} \parallel \overline{\Gamma\mathrm{M}'}$) shifts toward higher energy as one moves away from the M' point along the k_y direction ($\mathbf{k} \parallel \overline{\mathrm{M'K}}$). This systematic shift provides direct visualization of the saddle point nature of the dispersion at the $\bar{\mathrm{M}}'$ point. The color scale in **c** represents photoemission intensity.

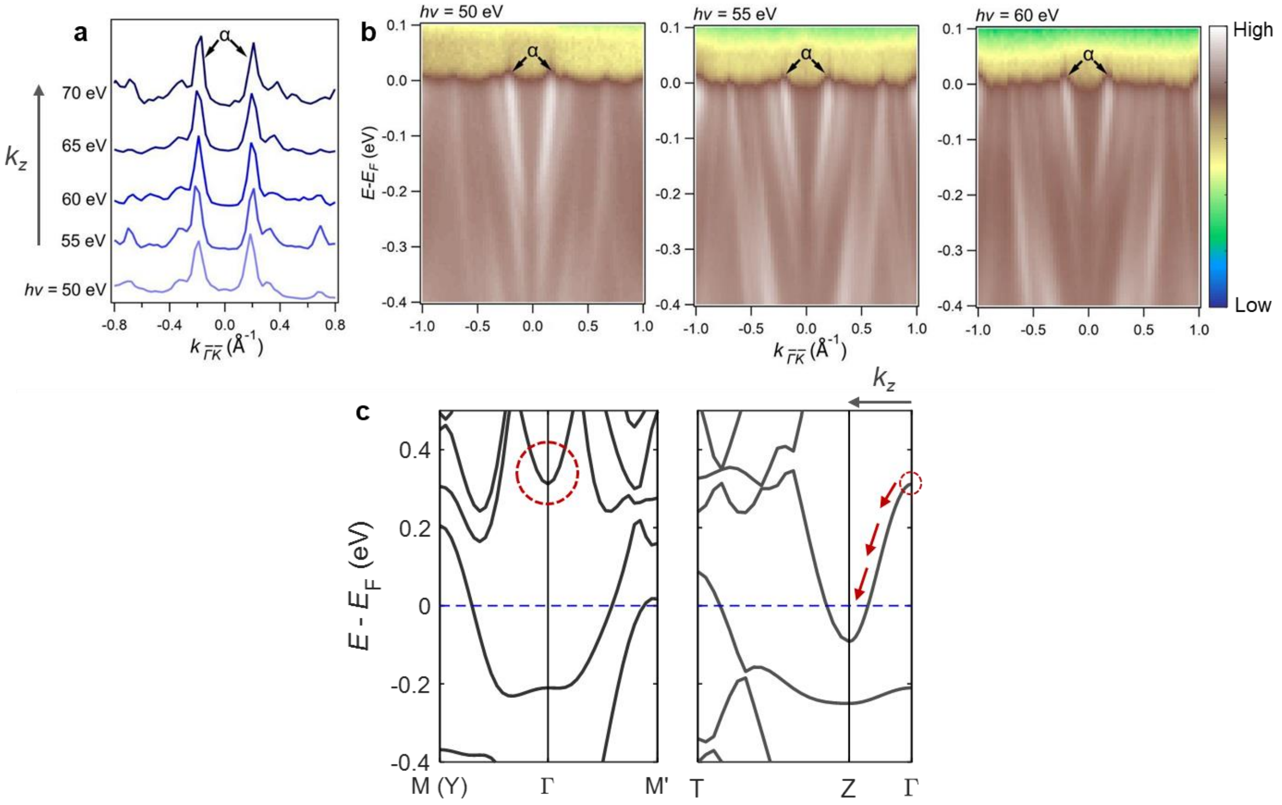

Supplementary Fig. 13 | A more detailed investigation into the nature of the α band. a–b, Examination of the α band based on its dependence on incident photon energy. Panel **a** shows stacked momentum distribution curves (MDCs) along the surface $\bar{\Gamma}\bar{K}$ direction at the Fermi energy for photon energies ranging from 50 to 70 eV, where the α band appears as distinct peaks around $k \approx \pm 0.2$ Å^{-1}. Panel **b** presents energy-momentum maps for 50, 55, and 60 eV photon energies, with the color scale representing photoemission intensity. **c**, DFT calculation results indicating a potential α band candidate, predicted to be present above E_F. The right panel demonstrates its substantial dispersion along the out-of-plane (k_z) direction, which is inconsistent with the observed behavior in **a–b**.

Supplementary References

1 Das, D. *et al.* Magnetic field driven complex phase diagram of antiferromagnetic heavy-fermion superconductor Ce3PtIn11. *Scientific Reports* **8**, 16703 (2018).

2 Continentino, M. A., Medeiros, S. N. d., Orlando, M. T. D., Fontes, M. B. & Baggio-Saitovitch, E. M. Anisotropic quantum critical behavior in $CeCoGe_{3-x}Si_x$. *Physical Review B* **64**, 012404 (2001).

3 Stewart, G. R. Heavy-fermion systems. *Reviews of Modern Physics* **56**, 755-787 (1984).

4 Coleman, P. & Nevidomskyy, A. H. Frustration and the Kondo effect in heavy fermion materials. *Journal of Low Temperature Physics* **161**, 182-202 (2010).

5 Ortiz, B. R. *et al.* Evolution of Highly Anisotropic Magnetism in the Titanium-Based Kagome Metals LnTi3Bi4 (Ln: La···Gd3+, Eu2+, Yb2+). *Chemistry of Materials* **35**, 9756-9773 (2023).

6 Guo, J. *et al.* Tunable magnetism and band structure in kagome materials RETi3Bi4 family with weak interlayer interactions. *Science bulletin* **69**, 2660-2664 (2024).

7 Bouaziz, J., Bihlmayer, G., Patrick, C. E., Staunton, J. B. & Blügel, S. Origin of incommensurate magnetic order in the *R*AlSi magnetic Weyl semimetals (*R* = Pr, Nd, Sm). *Physical Review B* **109**, L201108 (2024).

8 Hu, Y. *et al.* Magnetic coupled electronic landscape in bilayer-distorted titanium-based kagome metals. *Physical Review B* **110**, L121114 (2024).

9 Fisher, M. E. Relation between the specific heat and susceptibility of an antiferromagnet. *The Philosophical Magazine: A Journal of Theoretical Experimental and Applied Physics* **7**, 1731-1743 (1962).
