# Supplementary material for: Spin density wave and van Hove singularity in the kagome metal CeTi3Bi4
Source: Nat Commun. 2025 May 12;16:4384. doi: 10.1038/s41467-025-59460-4 (PMC12069633; doi:10.1038/s41467-025-59460-4)
Supplement: Supplementary file 2 — Description of Additional Supplementary Files [file 41467_2025_59460_MOESM2_ESM.pdf]

Description of Additional Supplementary Files

File name: Supplementary Data 1
Description: Optimized crystal structure of $LaTi_3Bi_4$ obtained from density functional theory (DFT) calculations.
